# The impacts of corporatisation of healthcare on medical practice and professionals in Maharashtra, India

**DOI:** 10.1136/bmjgh-2019-002026

**Published:** 2020-02-11

**Authors:** Shweta Marathe, Benjamin M Hunter, Indira Chakravarthi, Abhay Shukla, Susan F Murray

**Affiliations:** 1 SATHI, Pune, India; 2 Department of International Development, King’s College London, London, UK

**Keywords:** health systems, health policy, qualitative study

## Abstract

A heterogeneous private sector dominates healthcare provision in many middle-income countries. In India, the contemporary period has seen this sector undergo corporatisation processes characterised by emergence of large private hospitals and the takeover of medium-sized and charitable hospitals by corporate entities. Little is known about the operations of these private providers and the effects on healthcare professions as employment shifts from practitioner-owned small and medium hospitals to larger corporate settings. This article uses data from a mixed-methods study in two large cities in Maharashtra, India, to consider the implications of these contemporary changes for the medical profession. Data were collected from semistructured interviews with 43 respondents who have detailed knowledge of healthcare in Maharashtra and from a witness seminar on the topic of transformation in Maharashtra’s healthcare system. Transcripts from the interviews and witness seminar were analysed thematically through a combination of deductive and inductive approaches. Our findings point to a restructuring of medical practice in Maharashtra as training shifts towards private education and employment to those corporate hospitals. The latter is fuelled by substantial personal indebtedness, dwindling appeal of government employment, reduced opportunities to work in smaller private facilities and the perceived benefits of work in larger providers. We describe a ‘reprofessionalisation’ of medicine encompassing changes in employment relations, performance targets and constraints placed on professional autonomy within the private healthcare sector that is accompanied by trends in cost inflation, medical malpractice, and distrust in doctor-patient relationships. The accompanying ‘restratification’ within this part of the profession affords prestige and influence to ‘star doctors’ while eroding the status and opportunity for young and early career doctors. The research raises important questions about the role that government and medical professionals’ bodies can, and should, play in contemporary transformation of private healthcare and the implications of these trends for health systems more broadly.

**Key questions**

**What is already known?**

- There is an expanding and diverse private healthcare sector in many middle-income country settings; however, structural changes and resultant practices within the sector are poorly documented.
- Many parts of India have a burgeoning corporate healthcare sector and its growth has been accompanied by concerns about cash-for-referral practices, overtesting and overtreatment, and also violence against healthcare professions.

**What are the new findings?**

- Employment in larger facilities, notably corporate hospitals, is taking over from earlier models of employment for doctors in Maharashtra such as government employment and doctor-owner facilities.
- This is accompanied by changes in employment relations and widespread use of performance targets, which place new constraints on professional autonomy, particularly for early career doctors.
- Doctors and other respondents report that these changes are accompanied by the inflated cost of healthcare and exacerbate medical malpractice and distrust in doctor-patient relationships.

**What do the new findings imply?**

- Governments and medical professionals’ bodies must pay attention to ongoing structural changes in the private healthcare sector, the growth of corporate ways of working and the implications for medical practices and standards.

## Introduction

‘*Perhaps the most subtle loss of autonomy for the profession will take place because of increasing corporate influence over the rules and standards of medical work.*’ 1

Starr’s prediction referred to a transformation of healthcare that was in process in the USA in the 1980s, but as corporate organisations now are becoming increasingly visible players in healthcare systems in middle-income countries, his statement invites investigation in these new settings. In this article, we examine the contemporary experiences of doctors in India as they encounter these new forms of commercialised healthcare delivery in the large cities and consider the implications these have for their work and for the Indian medical profession.

In the latest phase of commercialisation of healthcare, development finance and private equity investments have together fuelled the growth of corporate hospitals in low-income and middle-income countries (LMICs).^2–6^ Large multispecialty and superspecialty private hospitals offer a new model of care provision but beyond that, as we argue in detail elsewhere, corporate ways of working also impact on the management and operation of medium-scale hospitals and charitable trust hospitals within the private provision sector.^7^ In line with the global push to expand the healthcare industry supported by the World Bank’s International Finance Corporation and other development financing institutions, healthcare is being viewed as providing potentially lucrative business opportunities.^8^ However, a recent Lancet series highlighted how little is known about the operation of private providers and the difficulty in assessing their effects on healthcare.^9 10^

This reorientation of healthcare also has significant implications for the working lives of those in the workforce of healthcare organisations. Various writers on transformation in the private healthcare sector have recommended more research on its effects on doctors and on medical practice.^11–14^ The literature on the effects of healthcare corporatisation on employment practices in high income countries emerged in the 1970s and 1980s^1 11 15 16^ and has been subject to renewed interest in the last few years.^17^

Research in LMICs has centred on the effects of private sector growth, including physicians’ views on the risks and benefits of the private healthcare sector in Vietnam and Ethiopia.^18 19^ In India, there have been claims that recent transformations in the private healthcare sector are encouraging the retention of health workers within the country,^20 21^ yet others point to incentives for medical malpractice.^20 22–24^ There are concerns regarding erosion of status for the medical profession as medical students and recent graduates face a series of challenges to complete their studies and find work, including indebtedness and the pressures of finding work in highly competitive fields,^21 25 26^ and senior doctors have noted the need for stronger support from medical associations for doctors working in the private healthcare sector.^22^ Wilson’s ethnographic study in the south Indian state of Kerala situates these challenges within a broader social transformation in which there appears to be a widening gulf between the high social status ascribed to the medical profession and the realities for young aspirants trying to establish medical careers, a point we will return to later.^27^

Here, we draw on the accounts of practitioners and those managing or observing their practice to provide an analysis of the contemporary situation of doctors trying to navigate the changing private healthcare sector in the large urban centres of Maharashtra state—as employment shifts from practitioner-owned small and medium hospitals to larger corporatised settings with elaborate management structures. In the first part of the findings section, we document changes in employment and consequent inequality in employment status. In the second part, we explore the implications of managerial and financial pressures for clinical practices. In the discussion section, we highlight the health systems implications of these findings and consider the implications for the medical profession with reference to recent sociological work on the changing professions.

This study was conducted in two large cities in the Indian state of Maharashtra, which has an estimated 4500 hospitals. Mumbai is Maharashtra’s largest city and India’s financial capital. Pune, the second largest city in the state, is a centre for the IT services industry. Seen as ‘medical hubs’ for both the country and the state, these cities have seen significant change in their healthcare systems since independence, characterised by: increasing pressures on an under-resourced public healthcare system; the emergence of small-sized and medium-sized private hospitals and, most recently, corporatisation trends involving growth of corporate hospitals, partnering of not-for-profit hospitals with management companies and erosion of the small-sized private hospitals.^7^ The national context for these changes is one of fast-growing economic activity in the healthcare sector, enabled by supportive public policy-making (see table 1), and including an influx of foreign direct investment estimated at US Dollars (US$) 6 billion since 2000 and a hospital industry expected to double in value between 2017 and 2022: from US$62 billion to US$133 billion.^28^

**Table 1:** Key milestones in the expansion of India’s corporate healthcare providers

Year	Event
1946–1980	Publication of the Bhore committee report emphasising universal coverage through a state-run health service. The federal government’s Five-Year Plans continue support for this goal
1983	National Health Policy, initiated expansion of public primary care, but promoted private sector for curative care
1983	Apollo Hospitals opens its first facility, later becoming the first publicly listed hospital company in India
1990	Tariffs on medical equipment imports were lifted and the insurance sector was opened to private investment
1998	Formation of Indian Healthcare Federation as an industry association to represent the formal private healthcare sector
1999–2005	World Bank sponsors a series of state-level Health Systems Development Projects which included support for public-private partnerships
2000	Restrictions on FDI lifted to allow 100% FDI for health-related services under the direct route
2000	IRDA opens up the market with the invitation for registration applications.
Early 2000s	Establishment of several large hospitals that would go on to become corporate chains, including Narayana, Fortis and Max. Concurrent decline in small, individual-run private healthcare providers
2002	National Health Policy sets out a framework that is broadly supportive of expanded private healthcare provision
2008	National government launches Rashtriya Swasthya Bima Yojana—a health insurance scheme for the informal sector which purchases healthcare services from public and private providers
2017	National Health Policy seeks to use strategic purchasing to align growth of private healthcare with public health goals
2018	A second national health insurance scheme, Ayushman Bharat, is launched by the government with increased cover for users

FDI, foreign direct investment; IRDA, The Insurance Regulatory and Development Act.

Medical doctors comprise 40% of India’s health workforce,^29^ are key players in healthcare provisioning and are often considered to be decisive in shaping healthcare practice. Approximately three-quarters of doctors are allopathic practitioners, the remainder are trained in ayurvedic and other alternative forms of medicine. Around 64 000 doctors with Bachelor of Medicine Bachelor of Surgery (MBBS) degrees^30^ and an overwhelming majority of students (91%) now wish to pursue a postgraduate specialist degree;^31 32^ however, each year, only 26 000 manage this.^30^ Their training is increasingly being provided by non-government institutions: according to the now-defunct Medical Council of India, there were 100 government medical colleges and 10 private medical colleges nationally in 1980; by 2019, the number of government schools had doubled while the number of private medical institutes increased 20-fold.^33^ Subsequent employment for doctors is concentrated in the private healthcare sector, where 90% of doctors in India are now based.^34^ Some commentators have pointed to the increased opportunities and monetary benefits offered to doctors by this trend,^22^ while others have highlighted concerns with a system that places significant constraints on clinical practice.^23^ There is widespread concern about unethical conduct, medical negligence, irrational treatment and overcharging by India’s private healthcare sector^24^ and frustrations among patients and their families frequently spill over into violence against healthcare workers. There are also growing calls from alliances of healthcare professionals and civil society for the prioritisation of ‘ethical’ approaches in healthcare.^35 36^

## Methods

### Data collection and analysis

The data reported here come from a larger mixed-method study on practices, regulations in and implications of the evolving private healthcare sector in Maharashtra State, India. Semistructured audio-recorded qualitative interviews were conducted between December 2017 and June 2018 with 43 respondents (table 2). We sought interviews representing a range of backgrounds, institutions and viewpoints and respondents were selected using a combination of purposive and snowball sampling. They were informed of the aims of the research and the purpose of the interview and were invited to participate in the research at a time and place of their choosing. At the time of the interview, respondents were informed how the data they provided would be managed, asked to complete a consent form and informed that they could withdraw their responses from the study at any time up until the end of the data collection period. Interviews were conducted by two of the authors (SM and IC), primarily in English and also in Hindi or Marathi as appropriate. Interview were questions related to changes in the practices of workers, patients and managers in the private healthcare sector.

**Table 2:** Characteristics of interview respondents

Characteristics of respondents	Number	Details
Doctors in clinical practice	23	Including 20 specialists (gynaecology, orthopaedics, urology, pathology, paediatrics); two general practitioners; one general medicine. Years of experience: <10 years: 2; 10–19 years: 5; 20–29 years: 9; 30+ years: 7. Including 11 current/former owners of a small-medium sized facility
Nurse	1	Working in a corporate hospital
Administrator/managers in private hospitals	11	Including CEO and mid-level managers in corporate hospitals, and an insurance company administrator
Senior government ex-officials	2	Responsibility for government health services; government insurance schemes
Patients of private sector services	5	Including representatives from patient fora in Mumbai and Pune
Academic	1	Expert on region’s health systems
Total respondents	43	Male 31; female 12 For those working in healthcare facilities, main base institution: corporate hospital 13; charitable trust hospital 8; small-medium sized private facility 12

An audio-recorded witness seminar, which is a form of group oral history used to document the history of recent events, was conducted on changes in the private healthcare sector in Pune and Mumbai since the 1980s. Ten witnesses—senior doctors, hospital managers, policy-makers and health system researchers—participated in developing an account of transformations in the sector. Recordings from interviews and witness seminars were transcribed by contracted assistants and every transcript was checked for accuracy by one of the authors. Verbatim interview transcripts and the witness seminar transcript were coded and analysed thematically by two of the authors (SM and IC) with assistance from Nvivo software.^37^ Initial descriptive codes were derived by the whole research team from a reading of existing literature with additional codes arising from a close reading of the transcripts and discussion among the research team. This was followed by more fine-grained interpretive coding and analysis. Initial findings from the research were ‘sense-tested’ with project advisors who work in the private healthcare sector in Mumbai and Pune, and at preliminary dissemination consultations held in Mumbai.

### Patient and public involvement

Patients were not involved in the design or management of this research.

## Findings

### Changing healthcare provision and employment opportunity

Respondents highlighted broad trends in employment options in the healthcare sector as the predominant model of provision has moved from public hospitals, and small and medium privately owned hospitals (often referred to as ‘nursing homes’) to a burgeoning corporate sector. In the current period, government employment has dwindling appeal for many respondents who cited difficult working conditions in urban public hospitals, poor pay offered in government as compared with most private hospitals, little enthusiasm for postings to remote and under-equipped facilities and the public sector’s inability to absorb a growing number of graduates with specialist training.

Respondents described a shift in the infrastructure of the private sector away from the owner-practitioner and towards work in large hospitals either as full-time employees or as visiting consultants. The opportunities to work in or run a small hospital have been reducing in recent decades, and a senior surgeon described the low-budget private nursing homes as *‘a dying phenomenon’*. Respondents cited the challenges faced by small hospitals. These include the difficulty for small setups to meet all regulatory requirements related to hospital infrastructure such as separate staircases, overhead tanks and sprinklers in the premises. They are unable to match the range and sophistication of corporate hospitals’ services and to meet changing patient aspirations; few are equipped to handle insurance schemes and lose those patients. For doctor-owners, the options are to tie up with corporate entities, hand over to a hospital management company or to close down and then join a corporate hospital as a visiting consultant or ‘panel’ doctor.

Staffing agencies also now provide doctors—and nurses—‘on-demand’ to hospitals. It has become common for new medical graduates to approach such companies for employment. In order to withstand competition in a crowded field, doctors have also resorted to the use of online ‘doctor-discovery and booking’ platforms such as Practo, which have emerged in large cities to enable self-promotion to potential clients. To appear near the top of its list requires payment of extra charges, but helps increase one’s visibility in the market.

### Doctors in debt

Medical education and the costs of setting up a practice both push doctors into indebtedness. Increasing numbers of students aspire to do postgraduate specialisation to stay competitive in domestic markets, as well as to open opportunities for migration. As noted above, the number of institutions and places for government training has been surpassed by private colleges, where many students have to pay hefty admission fees that are largely unregulated. One respondent noted that fees for training for radiology can cost the equivalent of US$100 000, around 17 times higher than in government medical colleges. A common route for a medical graduate had been to set up a new practice using family savings or small loans. This path to employment is, however, increasingly difficult. Any attempt to open one’s own clinic or hospital requires a loan because of the high costs for land, infrastructure, equipment and other resources. Some respondents suggested that all these debts put doctors under pressure to exact maximum financial returns from medical practice and earn more in order to repay their loans as soon as possible.

### Emerging new forms of employment relations

Old ways into private practice are thus becoming difficult. As the price of land and technology has become prohibitively expensive, even the strategy of starting out one’s practice in a large residential apartment has been undermined by more stringent enforcement of government building regulations in recent years. On top of this, there is a saturated market for many areas of medical practice in Mumbai and Pune, as one ophthalmologist noted: *‘when I started to practice in the 1980s, there were about 30–35 ophthalmologists in the city and today the number in Pune is 500 plus’.* This scenario disadvantages new starters who lack the reputation and client-base of established practitioners. As a specialist explained, for many doctors now *‘there is no choice but to work in a big hospital’*.

For those who do find work in the larger, usually corporate, hospitals, there can be significant employment benefits on offer including regular pay, access to advanced technology and infrastructure and well-trained human resources. Doctors working with corporate hospitals do not need to make investments nor worry about issues that would otherwise occupy significant portions of their time as owner-practitioners such as staffing, administration and renewal of a facility licence. Instead, they can focus on clinical practice. Some doctors perceive that doctors' attachment to reputed, big hospitals with brand names also has a positive impact on their patient as big hospitals are assumed to employ the best doctors. There are personal aspects that are attractive too. Larger hospitals also have security guards within the hospital premises that provide better protection against violence from patients. Prestige and credibility is derived from work with a reputed hospital, and indeed it was noted that young male doctors considered being associated with branded hospitals as advantageous in attracting a future spouse.

However, there is widespread precarity in employment conditions in private healthcare, especially for young, early career doctors. Access to employment in larger hospitals is limited by the small number of unoccupied posts and hospitals’ preference for doctors trained in complementary medicine who are cheaper to employ and can perform many of the same tasks. The competition means that many doctors have little control within employment relations. It was noted by many respondents that managers are able to terminate the contracts of workers at short notice. Another respondent described being asked to pay a bond to a hospital that they would receive back only if they stayed at the hospital for a certain number of years. Those specialities and hospital departments that generate smaller revenue than expected face a constant struggle to justify their continued presence in the hospital. In some hospitals, full-time contracts are used which prohibit practising elsewhere and involve heavy workloads and low pay: described by our interviewees as ‘*exploiting*’ doctors and paying ‘*pennies*’.

Respondents reported a major disparity in pay between junior and senior doctors in corporate hospitals. For example, junior doctors may be paid 100 000 rupees per month (US$1400) while senior doctor pay can exceed 10 million rupees per month (US$140 000). Given increasing competition and difficulties in practicing as a medical doctor, some medical graduates are opting for different professions. After completing the MBBS, they take a Masters degree in business administration, law or hospital management and pursue careers in related industries like pharmaceuticals, health insurance or the medical devices industry. Further up the professional hierarchy, doctors with established reputations often avoid full-time contracts in order to practise across several hospitals and can generate greater income. For a small number of such doctors, corporatisation trends have proven very lucrative. The last 20 years have seen the ascension of the ‘star doctor’: renowned doctors who become a type of celebrity. Usually, surgeons can attract wealthy patients, command large fees, hold multiple appointments across several hospitals and often appear in the marketing materials. Respondents noted that hospitals will charge higher fees from patients in order to accommodate the increased cost of employing ‘star doctors’.

One member of management staff at a large hospital in Mumbai highlighted the value of such doctors to hospital marketing teams and the moves made to retain them, revealing that ‘*one senior doctor who gets a lot of VIPs has now become the trustee so that he won't leave that hospital’*. However, another respondent—a surgeon—was unsure how long this privileged position would continue, arguing that as large hospitals become more established brands in their own right, they will be less reliant on famous doctors and opt to hire cheaper alternatives.

### Performance targets: ‘Were you not convincing enough?’

The shift from smaller to larger private providers, and from clinical autonomy to following standardised plans and protocol as defined by corporate hospitals’ management, is evident. Opinion on whether it should be viewed as progress differed, with some respondents seeing this as replacement of a low-quality market segment by one with greater technology, life-saving capacity and transparent systems. However, many saw this as part of a broader shift away from a patient-oriented healthcare provision towards systems of revenue generation that, according to one small hospital owner, are *‘faceless, impersonalised and less accountable’*. For these respondents, family practice or small hospitals were much more connected with their patients. The incursion of commercial considerations into everyday practice in the form of performance targets was a particular area of concern for our respondents. Monthly targets for revenue generation or patient throughput are reportedly set for full-time as well as for visiting consultant and panel doctors at the time of their appointment. A doctor who misses their targets for two consecutive months receives a verbal or written memo, and contracts can be terminated if performance against these does not improve. At times of contract renewal, the commercial value of the doctor is assessed on the revenue they have generated for the hospital.

Respondents also informed us that the pressure of meeting targets increases as the end of the month approaches. The success rate of converting outpatients to inpatients by recommending a procedure or surgical treatment requiring admission has become significant for the meeting of organisational targets. As a gynaecologist described it, if the inpatient revenue they generate is proportionately far less than outpatient revenue ‘*then doctors are asked “why is it so? Were you not convincing enough?”’*

Large corporate hospitals build their reputation on the twin selling points of inpatient ‘hospitality’ and of use of advanced technology. This in turn contributes to irrationalities in medical care as prolonged hospitalisation and large batteries of tests become normalised. Unnecessary tests are performed to attain the targets needed to ensure the return on the purchase of costly equipment: *‘Because of targets, machines require 1000 patients or investigations per month. So, it definitely involves a lot of unnecessary investigations, a lot of treatment modalities’* (Ophthalmologist). This situation is compounded by growing use of private insurance to pay for healthcare and attitudes that see this insurance as legitimising irrational provision of care.

As our respondents acknowledged, financial imperatives are not new to the private sector in India, nor are they unique to larger hospitals, as smaller private providers also have to remain financially viable. However, the point was made that targets in small hospitals are limited to the owner-doctor’s requirements for meeting loan repayments and are not individually applied in that way to other doctors attached to it.

Participation in networks of cash-for-referrals, referred to as ‘cut practices’, has been longstanding within Maharashtra’s healthcare system, encouraged by the financial strains on smaller private providers. Now the incentives are for practitioners to by-pass smaller hospitals and refer their patients to specialists in the larger, corporate hospitals in search of more substantial commissions. In the words of one gynaecologist: ‘s*ince the corporate bill is much bigger even the crumbs are bigger’*. Respondents explained that refusal to engage in paying cash for referrals could prove particularly detrimental to younger doctors without an established reputation and who might otherwise struggle to attract patients, impacting in turn on their ability to repay debts.

### ‘Doctors are pawns’

While doctors’ pay and conditions seem generally better in larger, corporate hospitals than in smaller private facilities, there are accompanying behavioural expectations and significant reductions in autonomy for medical professionals. The growth of a healthcare management cadre is evident in burgeoning management departments, new managerial posts such as operating officers, executives and supervisors, and the widespread incorporation of business practices. Most managerial staff are the products of business management training institutions. Respondents felt these staff prioritised financial concerns of their institution over the realities of healthcare—in ways that left one urologist comparing such hospitals to patient ‘*factories*’. The comment from a retired state health official that *‘doctors are pawns, they are supposed to be the conduit to earn money’,* reflects a commonly voiced cynicism about the healthcare industry and its ways of working. Doctors also felt they must accommodate the priorities of hospital managers, as one radiologist noted: ‘*if you want to work with corporates you have to have a certain mindset… in short you have to keep the management happy’.* Senior consultants can express resistance to management decisions but do not have a ‘deciding say’ in the running of the hospital. Junior doctors have even less opportunity to do so.

The division of labour within wards in corporate hospitals allows little time for patient interaction with senior doctors as the majority of care is provided by junior doctors and nursing staff. This results in a loss of status: ‘*Patients interact with doctor as if doctor is the employee of the hospital and hence should give the services*’ (Gynaecologist). The growing use of standard treatment protocols introduced by hospitals and by health insurers was felt by many to be an additional constraint on professional autonomy. While some felt this helped to standardise prices and improve transparency for hospital bills, other respondents noted that it further eroded the autonomy of healthcare workers in providing discretionary fee waivers to patients considered unable to otherwise pay.

## Discussion

The analysis in this article aims to understand the experiences of doctors who are navigating a changing scenario of private healthcare delivery in Mumbai and Pune, as seen through their own eyes and those interacting with them. The specificity of our sampling allowed for valuable in-depth data collection but is also a limitation. First, our findings do not include the views of doctors who work only in the government facilities. Second, while our findings are likely to have salience in settings where similar structural changes are taking place in medical education and employment, the plausibility of their applicability to other groups and settings needs to take into account contextual differences.

Some of the accompanying shifts taking place in public healthcare institutions have been the subject of earlier research,^38^ and our focus here on the private healthcare sector comes at a time of renewed national and subnational policy interest in the expansion of insurance financing and private provision for healthcare.^39^ We found the in-depth qualitative interviews suitable for generating personal accounts on changes in private healthcare delivery and almost all respondents agreed to audio-recording. We also wished to set these within the backdrop of the recent history of the sector, and we found the witness seminar technique particularly useful for bringing together a collective narration of contemporary events that were not well documented elsewhere.^40^

Our findings suggest several dimensions for understanding the contradictions affecting medical professionals navigating a shifting employment scenario in the contemporary private healthcare sector in India. Here, we discuss these in relation to relevant other literature.

### Significant restructuring of employment for doctors

Study respondents point to a notable shift taking place in the avenues for doctors’ training and employment. Earlier models of either employment by government colleges and hospitals, or being a small or medium hospital owner or a solo practitioner are declining, while the trend of doctors working within corporate hospitals is increasing. This is borne out by other reports.^14 41^ Corporate hospitals have indeed become a favoured destination for doctors' employment. Our findings suggest factors which are pulling the doctors towards the corporate hospitals and pushing them away from the small hospitals and public healthcare system, and point to the reduced choices for young and early career doctors beyond joining corporate hospitals. This is not confined to Maharashtra—reports from the south of India also indicate the shutting down of smaller clinics as doctors take offers of higher pay in corporate hospitals with which small facilities cannot compete.^42^

### Consequent changes in doctors’ relationship with their patients

An erosion of trust in the doctor-patient relationship has been noted in studies from India and other LMICs.^43 44^ Other studies have also shown that the perception of malpractice by the doctor for financial gain, or the impression that the doctor has neglected their duty towards patients and relatives are important contributors to aggression, leading to violence against doctors.^45^ In healthcare, the inter-relations of systems trust and personal trust are complex.^44^ In the context of the USA, commentators have for some time noted tensions between practitioner self-interest and notions of ‘collective altruism’ to serve the needs of patients, and the shift away from earlier paternalistic relationships towards more consumerist relationships.^46^

What our findings suggest is that doctor-patient relationships in the context of private healthcare in India are being influenced by three inter-related trends: changes in attitudes of doctors, practices in a corporatising hospital sector and aspirations among patients. Until the 1980s, medical practice was dominated by general practitioners and family physicians, where the doctor-patient relationship was personalised with an assurance of continuity of care. Information asymmetry meant patients were open to exploitation and unnecessary intervention, but as doctors were paid by individual families, this provided a degree of self-regulation due to a need to maintain a web of social networks and trust of patients. The transaction between the private general practitioner and patient, though commercial in nature was thus also relational and socially embedded.

Our respondents’ accounts suggest that corporate healthcare chains with aggressive marketing to develop client pools are operating as an explicitly commercial and socially disembedded enterprise and that their business practices exacerbate the risk of over-intervention. The corporate chains are part of a ‘medical-industrial complex’ that brings together the medical profession, politicians, religious organisations, financial capital, real estate, insurance and other non-health industries, and which is influential in public policy processes for pharmaceuticals, medical devices and healthcare provisioning.^14 20 47^

As doctors are paid by the corporate hospitals and not directly by patients, this also changes the patient’s relationship with their doctor. The focus of our study was not patients, but our doctor interviewees described how, with reduction in the number of small hospitals,^41^ the corporate hospitals’ empanelment with insurance companies, and the widespread publicity around high technology interventions, upper-middle-class patients’ preferences have gradually shifted from having a known and trusted doctor to the well-equipped, corporate hospitals with hotel-style facilities. These then set the aspirations of others.

### An emerging reprofessionalisation and restratification

For our final point, we return to consider Starr’s suggestion that increasing corporate influence over the rules and standards of medical work engender *‘perhaps the most subtle loss of autonomy for the profession’* 1 and how this is enacted in India. The literature suggests that the behaviours of other large health industries including insurance, pharmaceuticals and medical technology are all contributing in different ways to reduced autonomy within medical practice.^48–50^ Our findings indicate that the level of doctors’ individual autonomy diminishes as their stake in the ownership of the private facility reduces, from operating their own practice or facility to working in a corporate hospital. As a result of corporate involvement, the medical profession in India is also now increasingly subject to new forms of management with new ways of thinking about the business of medicine. Doctors report finding their clinical decision-making swayed by employer-imposed targets and driven by institutional and personal needs to ensure financial returns. The performance targets imposed on doctors in this system further encourage malpractices such as exaggerated diagnoses and unnecessary therapeutic procedures,^24^ and patients pay the price of undergoing unwarranted procedures and inflated costs of healthcare. These are experiences that resonate with those of doctors in other, wealthier countries.^12 51^

Doctors are not, of course, one homogenous group. Middle-level doctors in private healthcare are paid well but are given performance targets in return which directly intrude on their professional autonomy. Young doctors at the start of their careers are in an even weaker position, often overworked and paid less. Such changes when observed in wealthy countries were interpreted by early researchers as deprofessionalisation—the loss of professionals’ autonomy and power to managers or consumers^51^—or even proletarianisation—a total loss of control over the conditions of work, as well as a severe reduction in compensation.^1^ However, recently sociologists have elaborated a more complex reading of the processes they were observing, describing these as reprofessionalisation: where professional practices, identities and boundaries are redefined by the expectations of corporations, managers and customers^52^ and restratification: the reordering of power within professions with the emergence of new professional elites and new hybrids which in other settings have often combined medical and managerial roles.^50 51 53 54^

These definitions elaborated by Waring^55^ provide a useful frame for our findings, which suggest that a process of reprofessionalisation is occurring within private medical practice in India in which changing forms of status, power and inequality have resulted from new expectations and pressures from the corporations, managers and service users of the private healthcare sector. A reordering of power can also be seen at the intraprofessional level as the balance shifts away from senior general practitioners towards specialists and super specialist-professional elites working in the corporate hospital sector. This exacerbates historical stratifications based on class and caste, as differences in who can enter the prestigious medical schools and afford postgraduate specialty training carry through into employment opportunities. The extent of the contemporary high profile for the ‘star doctor’ or ‘super doctor’ is perhaps peculiar to India^56^ and may be understood as the emergence of a new professional elite that is a hybrid of medical practitioner, businessman and celebrity philanthropist.

## Conclusion

It has been noted by other researchers from LMICs^15 16^ and high-income countries^1 8 13 14 56^ that the changing, and increasingly global, healthcare industry has significant implications for doctors and for their practice. In India, tensions are being felt by medical professionals with regard to opportunities and challenges in the contemporary healthcare system where growing public policy emphasis on private healthcare provision and on insurance modes of financing feeds into corporatisation processes. This empirical study contributes to understanding the effects of corporatisation of healthcare on doctors in middle-income countries like India. We evidence three interrelated processes of restructuring, reprofessionalisation and restratification which can be observed in this context and which have been occasioned or exacerbated by corporates’ practices.

The case study from Maharashtra reveals an ongoing reprofessionalisation within the private medical sector encompassing changes in employment relations through the advent of corporate hospitals, personal indebtedness, performance targets and constraints placed on professional autonomy and accompanied by trends in cost inflation, medical malpractice and distrust in doctor-patient relationships. Contiguously, a restratification taking place within India’s medical profession is one which appears to favour senior hospital specialists and ‘star doctors’ who contribute to the corporates’ brands and patient recruitment while the status of senior general practitioners has diminished. Our findings suggest that a new section of the medical fraternity with elite status holds authority and is able to maintain strategic positions in this situation, while a decline in status and autonomy is being experienced by a large section of doctors especially by those commencing their careers.

Overall, professional practices, identities and boundaries are being redefined by the expectations of corporate healthcare providers, their managers and their users. This raises important questions about the role that government and medical professionals’ bodies can and should play in such a scenario where the potential for ‘collective altruism’ is being actively eroded.
